# Mutations in H5N1 Influenza Virus Hemagglutinin that Confer Binding to Human Tracheal Airway Epithelium

**DOI:** 10.1371/journal.pone.0007836

**Published:** 2009-11-18

**Authors:** Guadalupe Ayora-Talavera, Holly Shelton, Margaret A. Scull, Junyuan Ren, Ian M. Jones, Raymond J. Pickles, Wendy S. Barclay

**Affiliations:** 1 Department of Virology, Imperial College London, London, United Kingdom; 2 Cystic Fibrosis/Pulmonary Research and Treatment Center, Department of Microbiology and Immunology, University of North Carolina at Chapel Hill, Chapel Hill, North Carolina, United States of America; 3 School of Biological Sciences, University of Reading, Whiteknights, Reading, United Kingdom; University of California San Francisco, United States of America

## Abstract

The emergence in 2009 of a swine-origin H1N1 influenza virus as the first pandemic of the 21st Century is a timely reminder of the international public health impact of influenza viruses, even those associated with mild disease. The widespread distribution of highly pathogenic H5N1 influenza virus in the avian population has spawned concern that it may give rise to a human influenza pandemic. The mortality rate associated with occasional human infection by H5N1 virus approximates 60%, suggesting that an H5N1 pandemic would be devastating to global health and economy. To date, the H5N1 virus has not acquired the propensity to transmit efficiently between humans. The reasons behind this are unclear, especially given the high mutation rate associated with influenza virus replication. Here we used a panel of recombinant H5 hemagglutinin (HA) variants to demonstrate the potential for H5 HA to bind human airway epithelium, the predominant target tissue for influenza virus infection and spread. While parental H5 HA exhibited limited binding to human tracheal epithelium, introduction of selected mutations converted the binding profile to that of a current human influenza strain HA. Strikingly, these amino-acid changes required multiple simultaneous mutations in the genomes of naturally occurring H5 isolates. Moreover, H5 HAs bearing intermediate sequences failed to bind airway tissues and likely represent mutations that are an evolutionary “dead end.” We conclude that, although genetic changes that adapt H5 to human airways can be demonstrated, they may not readily arise during natural virus replication. This genetic barrier limits the likelihood that current H5 viruses will originate a human pandemic.

## Introduction

Influenza pandemics occur periodically and their significant effect on society is aptly demonstrated by the current ongoing pandemic caused by a swine origin H1N1 subtype influenza virus that has thus far claimed nearly 2000 lives in the span of 6 months in mid 2009 [1]. Avian influenza H5N1 viruses have infected more than 400 people since 2003 but no sustained human-to-human transmission has occurred to date [2]. The reasons why H5N1 fails to transmit among humans remain unclear especially since high viral titres have been isolated from the nose and throat of H5N1 infected individuals [3]. The host barrier for avian influenza virus spread in humans is multigenic. Many H5N1 isolates already contain mutations in viral genes such as the PB2 component of the polymerase complex that confer enhanced replication capacity in human cells, but still this has not rendered them transmissible. Other viral genes such as the viral hemagglutintin protein (HA) are undoubtedly key to understanding the H5N1 virus' pandemic potential.

The viral hemagglutinin protein (HA) in part dictates host range and cellular tropism of influenza virus through its preference for different conformers of sialic acid (SA) on host cell glycans that mediate virus attachment and facilitate infection (reviewed by [4]). In previous studies, the distribution of SA in the human airway epithelium has been investigated using linkage-specific lectins as SA probes. These studies showed α2-6 SA expression was abundant in the upper respiratory tract whereas α2-3 SA was scarce [5]. In contrast, in the distal lung regions both α2-3 and 2–6 SA expression was significant [6], [7]. While the precise nature of the molecule(s) used by influenza virus as a receptor are not known, sialic acid residues in the α2-6 conformer are preferentially used by the HA proteins of human influenza viruses while avian isolate HAs prefer SA with α2-3 linkages [8]. Significant evidence exists that a switch in SA preference of the HA, from α2-3 to α2-6 linked SA, is a necessary adaptation step for viruses with avian HAs to gain the ability for human-to-human transmission. Previous influenza pandemics of 1918, 1957 and 1968 support this requirement as each of the respective HA genes, originating from avian-like viruses, mutated to increase binding preference for α2-6 SA [8], [9], [10]. Notably two amino acid mutations in the HA receptor binding site (RBS) were important during the evolution of each pandemic virus. Viral sequences from early isolates in the pandemics suggest adaptation occurred in a stepwise manner as isolates with just one of the two mutations have occasionally been found [10]. Further, transmissibility of the 1918 influenza virus between ferrets was lost after conversion of two amino acids in the receptor binding site of HA to those of avian H1N1 viruses, whereas mutation of a single amino acid produced a viable virus with intermediate transmissibility [9]. Recovered sequences from other sources of 1918 influenza viruses indicate that the intermediate mutations also circulated during that time [10]. Whether or not the current H5N1 viruses are capable of progressive mutations that switch their SA linkage preference is central to an accurate risk assessment of the potential of H5N1 to be the progenitor of a human influenza virus pandemic.

A wide range of mutations that switch receptor preference of HA proteins have been identified and include changes throughout the HA gene. Key amino acids at positions 226 and 228 (H3 HA numbering) affect SA preference of several subtypes including H2, H3, H4 and H9 [8], [11], [12]. On the other hand the H1 HAs that are human adapted bear changes at positions 190 and 225 relative to their avian precursors [9]. Mutations of the H3 HA at position 190, 193 and 194 were all previously shown to alter SA preference [13], [14]. Changes in HA that affect the extent of its glycosylation can also profoundly affect the SA binding affinity and in vivo this may contribute to changes in tropism and pathogenicity [15], [16]. A selection of characterized mutations were previously engineered into recombinant H5 HA proteins and their effects on SA binding studied with red blood cells from different species [17], [18] or using synthetic glycans which act as receptor mimetics [19], [20], [21]. None of these mutations when present in H5 HA conferred a switch from α2-3 SA binding to α2-6 SA binding using these assays. However, SA presentation on red blood cells or as glycan mimetics may not accurately represent SA expressed on the human airway epithelium luminal surface. In addition, the use of lectins as probes for SA that are utilized by influenza viruses has recently been questioned [22], [23]. Although lectins are highly specific for terminal linkages in glycopolymer chains they do not interact with adjacent carbohydrates. Conversely, influenza HA proteins have more complex interactions with sialylated glycans and binding of HA to SA are affected by glycan topology at the cell surface [24], [25]. Thus, lectins may detect SA that cannot be utilized as receptors for influenza viruses and HA proteins may bind more avidly to some sialylated receptors not readily detected by lectins. Tests to assess binding of H5 and mutants thereof in relation to host range receptor switching should therefore strive to use models that reflect the microenvironment of the luminal surface of the human airway epithelium.

The human airway epithelium is a well-differentiated tissue consisting of ciliated cells and mucin-secreting cells that overlay a basal cell layer. *In vitro* models of human ciliated airway epithelium (HAE) that recapitulates the morphology, biochemistry and physiology of the epithelium are available. Such models have been used to study virus-host cell interactions for a number of respiratory virus including human and avian influenza viruses [15], [26], [27], [28]. In particular, we and others have shown that human and avian influenza viruses target different cell types in this model, a tropism related to the specific SA linkage displayed on these cells [15], [29], [30]. Recent data also suggest that the differential tropism in HAE cultures may account for the compromised spread of avian influenza viruses in the cooler conditions of the human upper respiratory tract[31]. In this model, avian influenza viruses exclusively infect ciliated cells whereas human influenza viruses that showed a switch in receptor preference to predominately α2-6 SA displayed an expanded cell tropism where both ciliated cells and non-ciliated columnar cells were infected. In addition, preferential binding of the H1 HA of the 1918 human influenza virus to non-ciliated cells of human tracheal tissue sections was recently reported [25].

Here, we use HAE cultures and tissue sections of human tracheal epithelium to determine the binding characteristics of wild-type and select mutations of H5 HA to human airway epithelium. In contrast to results published with short synthetic sialylated ligands, we show that mutations in H5 HA at residue 228 effectively switch the binding pattern from an avian-like HA to that shown by the HA of a human influenza H3 virus. Detailed sequence analysis of codon usage at this site by H5 revealed that although this mutation is possible, currently circulating H5N1 viruses are not poised to acquire this amino acid change easily. Our findings confirm the potential for H5 to adapt to human airway epithelial cell receptors but suggest that it is unlikely to occur within the quasi-species present in an infected individual. Our data offers a rationale for why H5N1 viruses have not yet evolved into human transmissible strains with pandemic potential.

## Results

### Expression and glycan binding of synthetic H5 HA proteins

**Figure 1:**
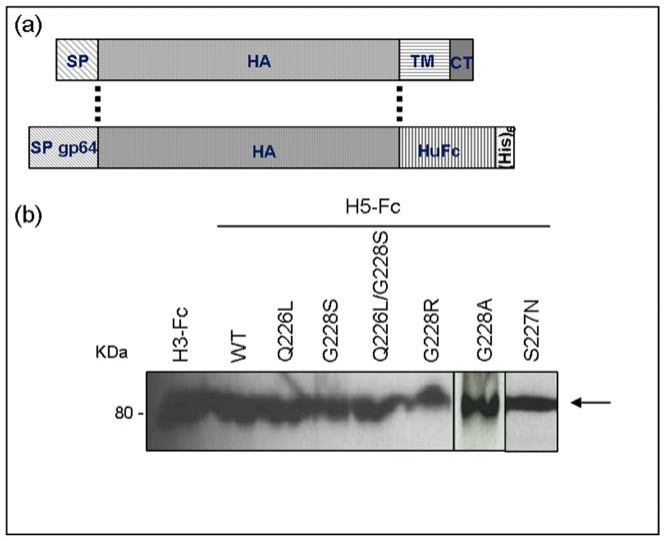
Expression of recombinant HA-Fc proteins. (A) Recombinant baculoviruses encoding HA from a recent H3N2 human virus, (A/Panama/2007/99) or from a highly pathogenic H5N1 avian influenza virus (A/Vietnam/1194/04), were generated as previously described [32]. The HA proteins were expressed as soluble proteins secreted from infected *Sf9* cells by removing the HA transmembrane (TM) and cytoplasmic tail (CT) portions of the protein and replacing the HA signal peptide (SP) with the signal peptide of the baculovirus envelope protein gp64 (SP gp64). The HA proteins were tagged at the C-terminus by a human Fc (HuFc) and hexa-histidine (His6) tags. (B) All recombinant HA proteins were expressed at similar levels as determined by western blot analysis using an anti-human Fc antibody.

**Figure 2:**
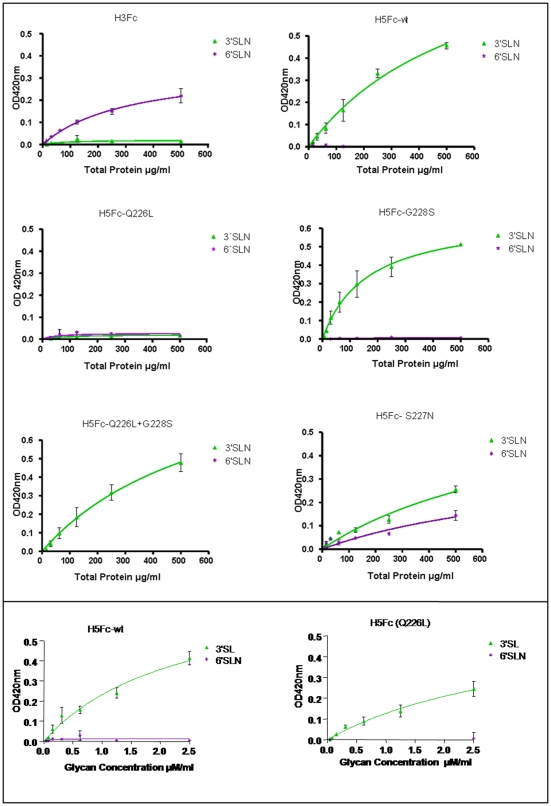
Binding of recombinant HA proteins to synthetic receptor ligands 3SLN (avian receptor) and 6SLN (human receptor) in a solid phase assay. (A) Synthetic glycans on polyacrylamide linkers were immobilized on 96-well plates following UV treatment. Recombinant HA proteins were preformed into higher order complexes by incubation with anti-human Fc before incubation on the plate and detection with goat anti-human IgG conjugated to horse-radish peroxidise (HRP). (B) Recombinant HA-Fc proteins were adsorbed on 96 well plates coated with anti-human Fc antibody. Synthetic glycans on polyacrylamide linkers with biotin tags (6SLN and 3SL) were incubated on the plates and detected with Streptavidin – HRP conjugate.

We generated recombinant wild-type and mutant H5 HAs and used them as probes to determine their ability to bind to SA on the luminal surface of human airway epithelium. For our wild-type H5 HA we chose the A/Vietnam/1194/04 H5N1 isolate (accession number: AY651333), as this virus was isolated from an infected human lung but was not associated with human-to-human transmission. Wild-type H5 HA was cloned as a fusion protein with the Fc domain of human immunoglobulin heavy chain as a immunological tag and expressed as a soluble molecule in the supernatant of recombinant baculovirus-infected insect cells (Figure 1A and [32]). We then introduced either single or double mutations at Q226L and G228S in the SA binding site of H5 HA [21] equivalent to those previously shown to switch receptor specificity in H2 and H3 HA subtypes [8]. Our previous work has shown these mutations alter red blood cell binding by H5 HA [18]. Equivalent mutations have not been documented in H5N1 viruses isolated in nature. For comparison, we also generated a S227N mutant identified in several human H5N1 virus isolates and reported to show changes in SA binding profiles in synthetic glycan assays [33]. All HA mutants were similarly expressed using recombinant baculoviruses and were secreted from infected *Sf*9 cells at similar level to the wild-type H5 HA protein [32] (Figure 1B). A solid phase binding assay was used to evaluate the binding of the HA-Fc proteins to short synthetic receptor mimics bearing a single copy of lactosamine with terminal SA in the α2-3 (3SLN) and α2-6 linkage (6SLN) (Figure 2) [32]. As expected, H5 HA bound to 3SLN and not to 6SLN whereas H3 HA cloned from the recently circulating human H3N2 virus (A/Panama/2007/99, accession number: CY034100) showed the opposite preference. Most of the H5 HA mutants retained affinity for the 3SLN glycopolymer. Interestingly, mutant Q226L did not bind to either of the sialyated lactosamine ligands tested in this assay. We therefore assayed Q226L H5 HA mutant protein for binding to the conventional α2-3SA lactose ligand, 3SL, since this has been reported by others [33]. In agreement with the previous report, we found that both wild type H5 HA protein and the Q226L mutant bound efficiently to the 3SL ligand confirming that the Q226L protein was correctly folded and represented a relevant ligand for our further studies. Of all the H5 HA mutants tested, only S227N acquired affinity for 6SLN in accordance with previous findings [33]. This mutant also retained 3SLN affinity, although it was reduced compared to that of the wild-type H5 HA.

### Binding of HA proteins to an in vitro model of human airway epithelium

**Figure 3:**
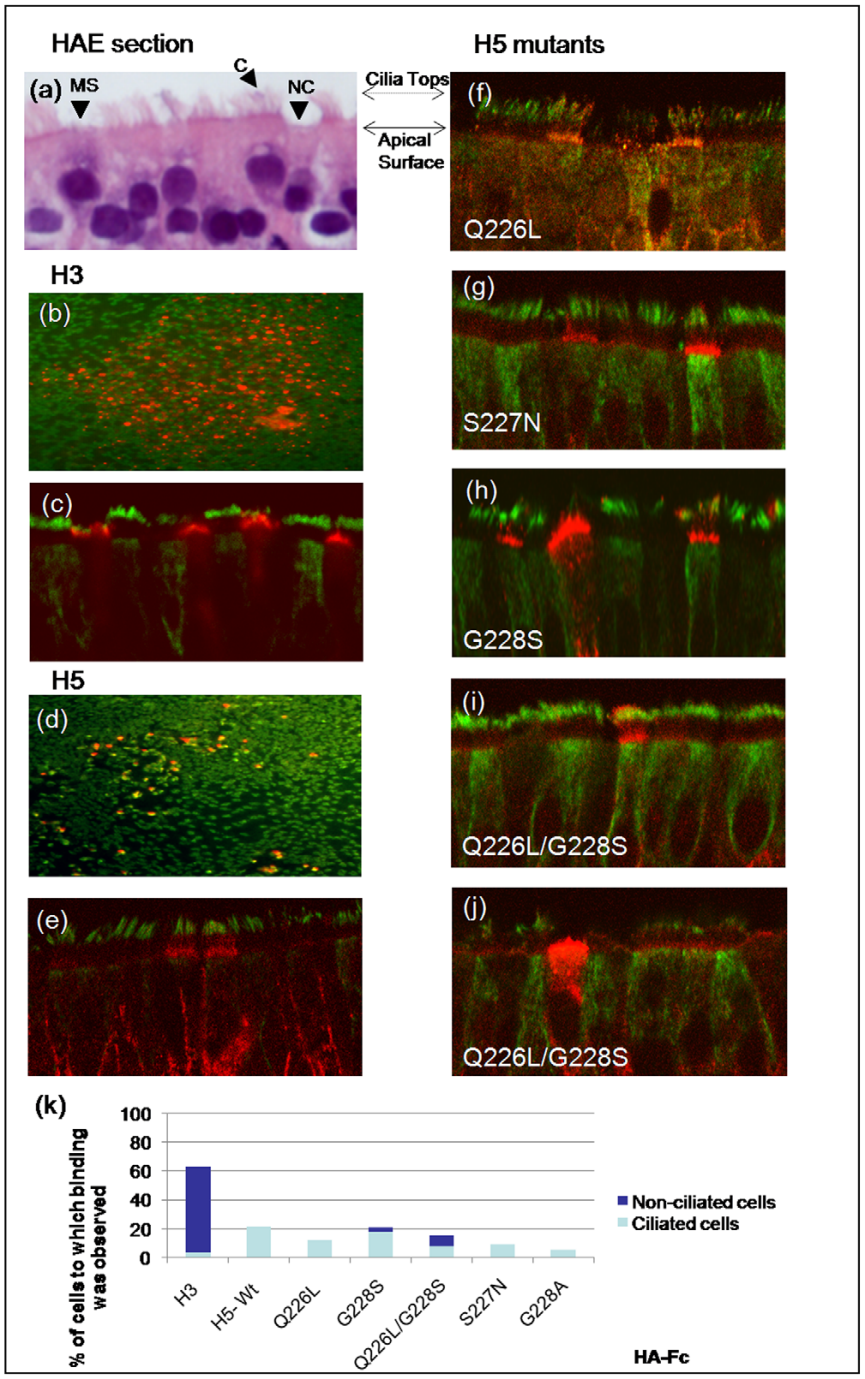
Recombinant HA proteins bind to human airway epithelial (HAE) cultures. (A) Morphological features of HAE cultures visualized by H&E counterstain. Cell types present include ciliated cells (C), mucin-secreting cells (MS) and non-ciliated cells (NC). HAE cultures were probed with recombinant HA proteins from human H3 (A/Panama/2007/99) (B,C) or avian H5 (A/Vietnam/1194/04) (D,E) viruses either directly to the apical surface of fixed cultures (B,D) or to histological sections (C,E). Receptor binding site mutants of H5 HA (F–J) were also analysed for binding to histological sections. Ciliated cells were identified using anti acetylated α-tubulin (green) and the HA-Fc proteins were visualized with anti human-Fc (red). Images are representative of cell-type binding seen in experiments. (K) Numbers and types of epithelial cells bound by HA proteins were quantified by counting 100–200 total cells from five different fields of view.

**Figure 4:**
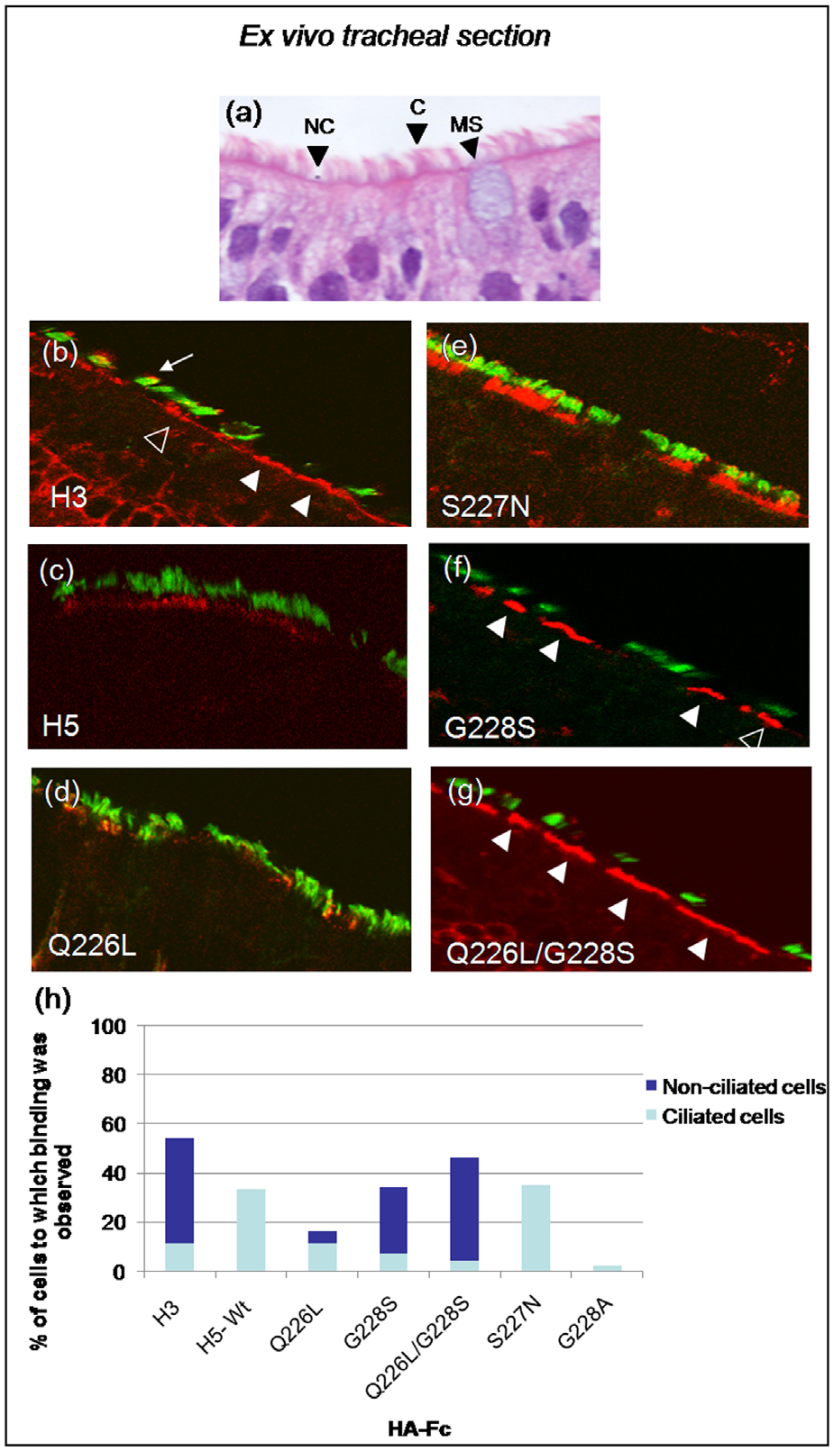
Recombinant HA proteins bind to human tracheal airway epithelium. (A) Morphological features of human tracheal airway epithelium ex vivo visualized by H&E counterstain indicating cell-types present: (C) ciliated cell, (NC) non-ciliated cell, (MS) mucin secreting cell. Histological sections of *ex vivo* human tracheal tissue were probed with recombinant HA proteins cloned from human H3 (B), or avian H5 (C) influenza viruses or with H5 HA receptor binding mutants (D–G). Ciliated cells are identified with anti acetylated α-tubulin (green) and the HA-Fc proteins detected with anti human-Fc (red). White arrows indicate cells that exhibit HA binding. Open arrowheads indicate ciliated cells with HA binding, solid arrowheads indicate non-ciliated cells with HA binding and the small arrow indicates binding to tops of cilia. These are representative images and similar patterns were seen in repeated experiments. (H) Numbers and types of epithelial cells bound by HA proteins were quantified by counting 100–200 total cells from five different fields of view.

The human airway epithelium, the predominant site of human influenza virus infection, contains a mixture of ciliated and non-ciliated epithelial cell types. Ciliated cells are abundant and can be identified morphologically by the presence of cilia and immunologically by antibodies directed against their expression of acetylated α-tubulin. Amongst the non-ciliated epithelial cell populations, pre-ciliated cells, mucin-secreting (Goblet cells), and other secretory cells may be present depending on the anatomical site examined (Figure 3A and Figure 4A). We and others have shown that using *in vitro* models of human ciliated airway epithelium (HAE), human influenza viruses infect both ciliated and non-ciliated cells, while avian influenza viruses infect only ciliated cells [15], [34]. In HAE, α2-3 SA expression detected by MAA lectin binding was evident at the tips of cilia and on the apical surface of ciliated cells likely at the level of the microvilli. MAA binding was only rarely seen on cells that lacked obvious cilia. In contrast, SNA lectin binding detected α2-6 SA on the apical surface of both ciliated and non-ciliated cells [15].

Since the recombinant HA-Fc proteins should represent more biologically relevant ligands than lectins for airway surface SAs utilized by influenza viruses, we used the HA-Fc fusion proteins to probe the apical surfaces of HAE, detecting HA binding with an anti-human IgG antibody conjugate. The dimeric HAs were pre-complexed into larger multimers by mixing with anti-human Fc antibodies before application to the tissue. When applied *en face*, H3 HA-Fc predominately bound to non-ciliated cells (Figure 3B), a finding that was confirmed when histological sections of HAE were similarly probed and immuno-reactivity for H3 HA-Fc localised predominately to the apical surface of non-ciliated cells (Figure 3C). In contrast, avian H5 HA-Fc bound sporadically to the apical surfaces of HAE and was only detected in association with cells that co-stained with acetylated α-tubulin, i.e., ciliated cells (Figure 3D and 3E). Binding of H5 HA was concentrated at the levels of the microvilli on ciliated cells as opposed to the shafts of cilia (Figure 3E), similar to the binding of the MAA lectin previously described [15].

H5 HA mutants Q226L and S227N bound to ciliated cells at the level of the microvilli similar to the parental avian H5 HA (Figure 3F and 3G). However, the G228S mutant showed an expanded binding profile that included binding to non-ciliated cell types in addition to ciliated cells (Figure 3H). The HA mutant bearing G228S in combination with the Q226L mutation also bound to both ciliated and non-ciliated cells (Figure 3I and 3J) indicating a partial switch in cell tropism.

The binding of each mutant to the different cell types was quantified by counting at least five different fields of view containing a total of 100–200 cells. Figure 3K shows that the H3 HA protein bound to approximately 60% of the HAE cells and the majority of those bound were of the non-ciliated type. In contrast all of the H5 HA proteins bound to fewer cells and of the cells bound the majority were ciliated although the mutants containing the G228S change bound to some cells that were non-ciliated (dark blue).

### Binding of HA proteins to human airway epithelium tissue sections

To determine if HA interaction with HAE cultures is predictive for human airway epithelium *in vivo* we next probed histological sections of freshly excised human tracheal airway tissue with recombinant HA-Fc. Similar cell types were present in the tracheal airway tissues as observed in HAE cultures confirming published data that HAE cultures recapitulate the morphology of the tracheal airway epithelium (Figure 4A). When HA-Fc were used to probe the human tracheal airway sections, H3 HA-Fc bound extensively to the luminal surface of the tissue and was localized at the apical surfaces of cells. H3 HA bound predominantly to non-ciliated cells in the tracheal sections, but also bound to some ciliated cells both at the level of microvilli as well as, in some cases, to the tips of the cilia (Figure 4B). In contrast, H5 HA-Fc bound only sporadically to ciliated cells at the level of microvilli (Figure 4C). The single mutants Q226L and S227N bound to ciliated cell microvillus regions (Figure 4D and 4E), similar to wild-type H5 HA (Figure 3E). In addition, some binding of the S227N mutant to cilia was observed (Figure 4E). In contrast, HA with the G228S single mutation bound to both ciliated cells and non-ciliated cells (Figure 4G). The combined Q226L/G228S mutation exhibited a strikingly similar binding pattern to that shown by the H3 HA, with binding predominately localized to the apical surface of non-ciliated cells (Figure 4G versus 4B).

Once again the extent of binding of each mutant to the different cell types was quantified by counting cells in at least five different fields of view (Figure 4H). This clearly demonstrated that the double mutant Q226L/G228S bound both to more cells in the section and showed an altered preference for the non-ciliated cell type in comparison with the wild type H5 HA. The extent of binding by G228S and the double mutant approached but did not reach the 55% of cells bound by the human adapted H3HA. Moreover although the S227N HA mutant showed some binding to the cilia tips (Figure 4E), analysis of several fields of cells obtained from different patients did not reveal any binding to the non-ciliated cell type, in contrast to the G228S containing mutants.

**Figure 5:**
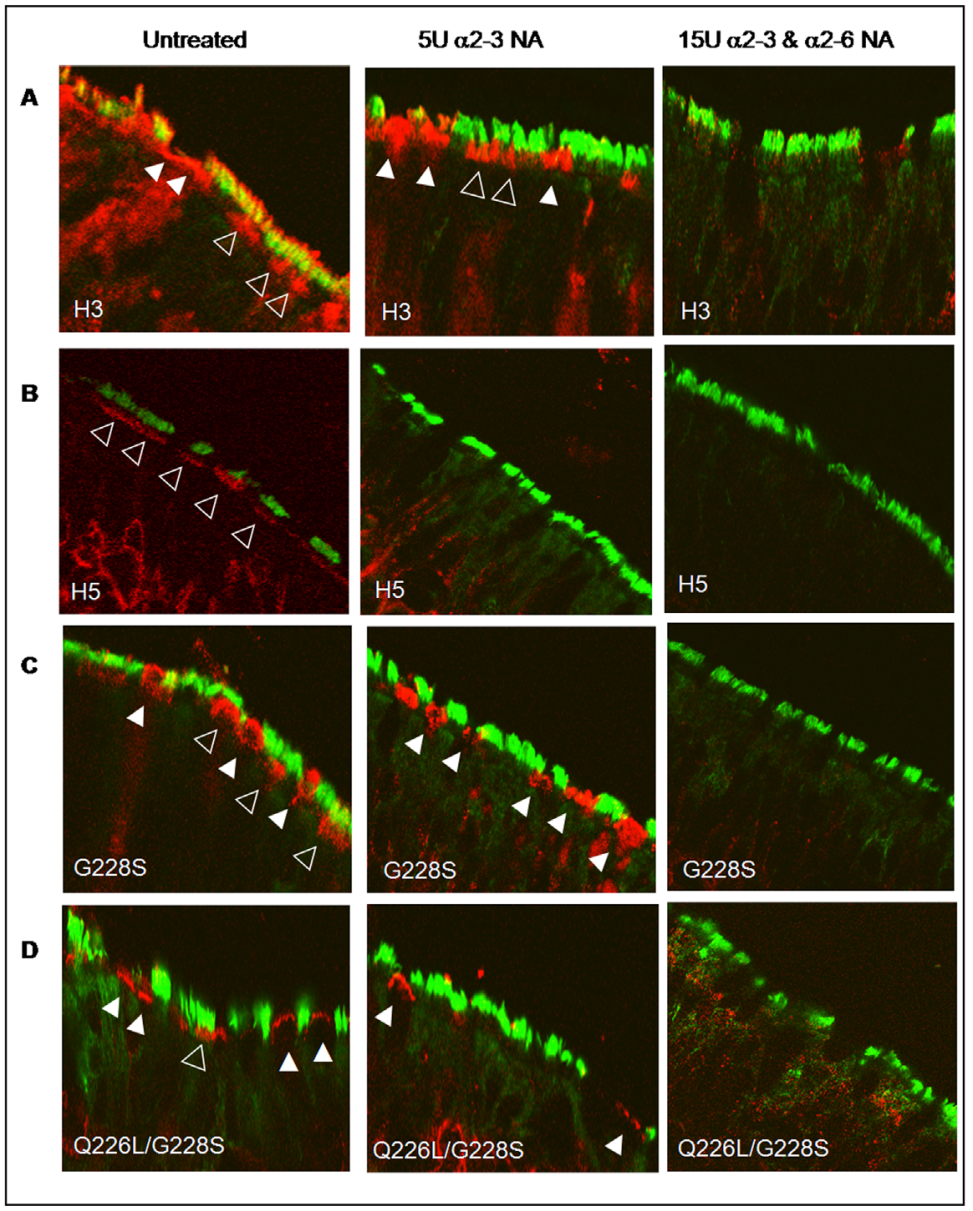
Recombinant HA proteins from human and avian influenza viruses bind *ex vivo* human tracheal epithelium in a sialic acid dependant manner. Histological sections of *ex vivo* human tracheal epithelium were probed with human H3 (A), avian H5 (B) or H5 mutants, (C) G228S, and (D) Q226L/G228S, with and without prior neuraminidase (NA) treatment. Slides were pre-incubated for 3 hours with 5 units of recombinant NA cloned from *Salmonella typhimurium* LT2 (NEBL) that shows a 260-fold preference for α2-3 over α2-6 linked SA or with 15 units of recombinant NA cloned from *Clostridium perfringens* (NEBL) that cleaves both α2-3 and α2-6 SA linkages. HA was detected as before using anti-human Fc (red) and anti-acetylated α-tubulin was used to indicate ciliated cells (green). White arrows indicate cells that exhibit HA binding. Open arrowheads indicate ciliated cells with HA binding; solid arrowheads indicate non-ciliated cells with HA binding.

To confirm that the binding of HA-Fc observed in these studies was SA dependent, tracheal airway sections were exposed to recombinant neuraminidase before probing with HA-Fc. Treatment with neuraminidase (NA) cloned from *Salmonella typhimurium* LT2, which displays a 260-fold substrate preference for α2-3 SA over α2-6 SA [35] abrogated binding of H5 HA-Fc to human trachea epithelium (Figure 5B, middle panel). Binding of H3 HA-Fc to the tops of cilia was also markedly reduced after treatment with the α2-3 specific NA, but binding to the apical surface of cells lacking cilia persisted (Figure 5A, middle panel). However, treatment with a broad specificity NA isolated from *Clostridium perfringens* abolished both the H3 and H5 HA binding (Figure 5A and 5B, right hand panel). The binding of the single or double mutants containing the G228S mutation on ciliated cells was abolished by treatment with α2-3-specific NA (compare Figure 5C and 5D, left panel, open triangles, with Figure 5C and 5D, middle panel). However, binding of these HA proteins to non-ciliated cells (solid triangles) remained unless the broad specificity NA from *C. perfringens* was used (Figure 5C and 5D, right panel).

### Analysis of H5 HA codon usage at amino acid 228

Our data show a specific amino acid mutation in H5 HA is sufficient to switch an avian virus-like binding profile (ciliated cells) to a human virus-like binding profile (ciliated and non-ciliated cells) in tracheal airway epithelium *in vitro* and *ex vivo*. The mutation enabling this event is a change in amino acid position 228 from G to S which likely increases the affinity of HA for the long chain α2-6 SA lactosaminoglycans in the previously reported ‘umbrella’ topology [21], [24].

**Figure 6:**
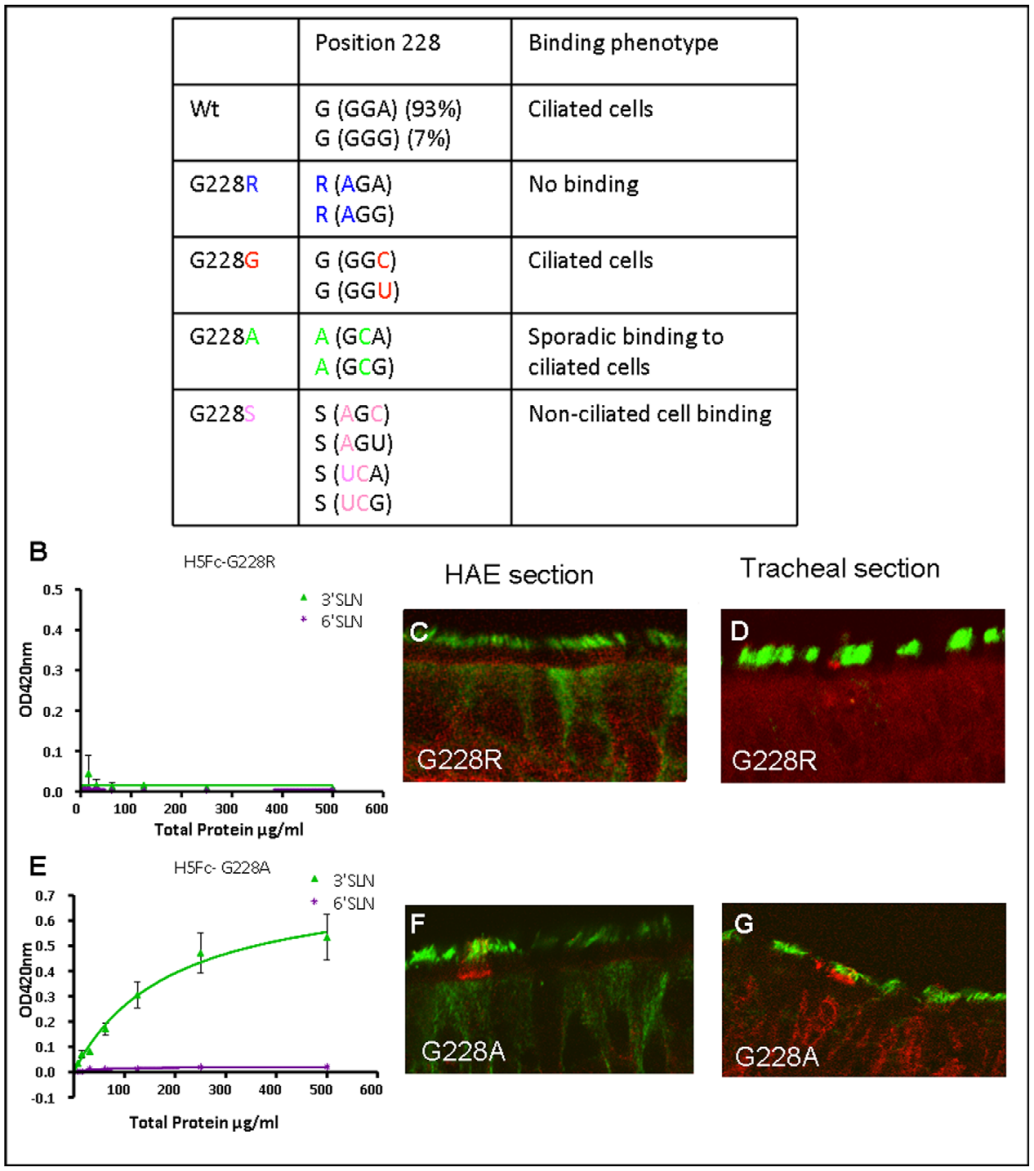
Nucleotide sequence changes required to switch H5 HA receptor binding preference. (A) Analysis of codon usage by 1374 H5 influenza viruses indicates that codon 228 is GGA in 93% strains and GGG in 7%. The table illustrates transversions and transitions by which coding capacity at this residue might change from glycine to serine. The ability of the two intermediate mutants 228R (B) and 228A (E) recombinant HA proteins to bind synthetic receptor ligands 3SLN (avian receptor) and 6SLN (human receptor) in a solid phase assay was assessed. The binding phenotype on HAE (C,F) or human tracheal epithelium (D,G) by recombinant proteins with each amino acid sequence changed at residue 228 are shown (C,D) G228R and (F,G) G228A.

While change of H5 HA specificity is experimentally possible, the propensity for the requisite mutations to occur *in vivo* depends on the feasibility of the circulating strains of H5N1 virus acquiring the necessary nucleotide changes. We assessed whether principles of codon usage would enable such a mutation to occur in the context of natural virus. An amino acid switch from glycine to serine at residue 228 requires either one or two nucleotide changes depending on the nature of the third ‘wobble’ nucleotide in the glycine codon. All of the 1374 influenza H5 HA gene sequences deposited in the available databases show that H5 viruses encode G228 using codons GGA (93%) or GGG (7%) (Figure 6A). Therefore, generation of a G228S mutation in H5 HA must involve one of three possibilities: 1) Simultaneous mutation of two nucleotides, the frequency of which is calculated to be lower than the maximum viral load of 10^8^ copies of vRNA would allow [3], assuming an error rate of 10^−5^ mutations per site [36]; 2) Transversion at the third nucleotide of the codon to generate a neutral intermediate that is unlikely to become enriched within the quasispecies; or, 3) Passage through an intermediate in which either the first nucleotide of the codon mutates initially to encode arginine by transition, or the second nucleotide of the codon mutates by transversion to encode alanine (Figure 6A). To assess the latter possibilities, we generated H5 HA-Fcs representing the G228R and G228A intermediates and tested their binding to synthetic glycopolymers, HAE and human tracheal airway tissue (Figure 6B–6G). Mutant G228A was unaffected in synthetic ligand binding compared to wild-type H5 HA (Figure 6E versus Figure 2) and bound sporadically to ciliated cells in HAE and tracheal tissue. Thus, this amino acid change would confer no advantage to a virus in the human respiratory tract. Mutant G228R failed to bind any of the synthetic ligands and did not bind any cells in HAE or tracheal tissue. Moreover, when the mutated H5 HA genes were used in attempts to rescue live influenza virus using reverse genetics we were unable to rescue viruses containing the G228R mutation. This is consistent with the notion that a G228R H5 HA is not capable of generating a viable intermediate during the evolution of a receptor switched virus. In contrast, viruses with wild-type H5 HA RBS or bearing other amino acid changes at residue 228 such as G228A or G228S were rescued (data not shown).

## Discussion

We address potential reasons why H5N1 avian influenza virus has not yet adapted for human-to-human transmission in spite of the unprecedented distribution and frequent zoonoses of this avian influenza virus. Text book explanations of the origins of influenza virus pandemics describe that reassortment of genes from avian strains and human strains give rise to pandemic viruses. One possible explanation why this event has not occurred for H5N1 could be an incompatibility between the H5 HA gene and other genes of currently circulating human influenza viruses. However, laboratory-generated recombinant viruses have shown that surface antigen genes from South East Asian H5N1 viruses are compatible with internal viral genes from currently circulating H3N2 human strains and coinfection of ferret in a laboratory setting produced *in vivo* reassortants with mixtures of genes derived from human circulating virus and H5N1 [37], [38]. Whether there is an impediment to the reassortment happening in nature is not resolved. In addition, an increasing body of work supports the notion that reassortment *per se* is not sufficient to generate a pandemic. Importantly, both unmodified H5N1 avian influenza viruses and laboratory generated chimeric human: avian reassortant viruses with unmodified H5N1 surface antigens failed to transmit between ferrets, the most relevant animal model for studies of human-like transmissibility of influenza viruses [39], [40]. Moreover, similar work with other avian subtypes of influenza virus indicate that mutations in the receptor binding site of HA that switch the SA preference are required for efficient transmission in the ferret model. For example, avian influenza viruses of the H7 subtype North American lineage that have undergone such a receptor switch showed transmission between ferrets in a direct contact model [41]. Similarly, H9N2 viruses whose HA genes display enhanced preference for α2-6SA binding were transmitted between co-housed ferrets whereas viruses with HAs that bound to α2-3SA were not [12].

By analogy with these other avian subtypes and in light of the experiences of previous pandemics [8], [9] we judge that adaptation of H5 HA at the RBS will be required for human-to-human transmissibility. Our data, summarized in Figure 6, provide a rational explanation of why H5 HA has not yet adapted in this way. By analysis of codon usage required to generate mutants capable of SA switching, we found that two or more simultaneous nucleotide changes are required to adapt the H5 HA gene for efficient binding to cells of the human tracheal epithelium. Moreover, our data indicate that single nucleotide change intermediates would be predicted to reduce viral fitness in the environment of the human airways. Indeed, attempts to generate such single nucleotide mutants in the context of infectious H5N1 virus indicate they are either non-viable or display attenuated growth kinetics (unpublished work).

Although some naturally occurring H5 HA mutants with increased affinity for synthetic human receptor mimics, including the S227N mutation considered here, have been detected within the quasispecies of viruses isolated from H5N1 infected patients [33], [42], [43], [44], none of them have resulted in documented human-to-human transmission of the virus suggesting that acquisition of binding to synthetic ligands is not an adequate predictor of transmissibility. Mutations spread throughout the HA protein have been found to affect receptor binding affinity and preference in the context of other HA subtypes [8], [9], [13], [14], [15], [20]. Although we cannot claim that the mutations we describe are the only ones that will switch receptor binding preference of H5 HA, extensive investigation of pandemic evolution [45] coupled with the cumulative research of several groups [18], [20] suggest they are the most likely candidates. Moreover, the failure of H5N1 to emerge as a pandemic strain despite the wide variety of species infected since 2003 suggests that there is a significant natural barrier to the evolution of H5 HA receptor binding specificity.

The reasons why HA of avian influenza virus has to adapt its RBS before acquiring human transmissibility are still not explained in detail. Studies utilizing lectin probes suggest a paucity of α2-3SA in the upper respiratory tract [9]. Our data shows that H5 HA protein can bind to α2-3SA on human airway epithelium but that the cell specificity of the binding differs from that shown by a human influenza HA. Interestingly the binding of H5 HA for synthetic α2-3sialylated glycans showed a different pattern than that shown for the human para-influenza viruses that also bind to the ciliated cell in human airway and transmit effectively between humans [27] Although replication of H5N1 virus in human nasal tissue *ex vivo* has been demonstrated [23], the scenario during actual transmission events between human hosts may be more limiting. In these cases, virus attachment might only occur to certain cell types that are in a specific anatomical location or that most abundantly display the relevant ligand.

Our findings that mutations in the H5 HA gene can alter receptor binding in the human airway epithelium may have implications for the generation of effective live attenuated vaccines. Although they are unlikely to be used during the pre-pandemic period due to the risk that reassortment with circulating strains might generate a novel virus with pandemic potential itself, live attenuated H5N1 vaccines may represent an important dose sparing strategy for wide scale vaccination of the population in the face of an ongoing pandemic with highly pathogenic H5N1 virus. Indeed, cold adapted influenza viruses with wild-type H5N1 surface antigens have been tested, but their immunogenicity in ferrets and in humans have been disappointing to date [46]. Using reverse genetics, the Q226L and G228S mutations could be artificially engineered in recombinant H5 HA within the cold adapted backbone. This may increase the vaccine virus' ability to replicate in the human upper respiratory tract and thus induce a more substantive immune response leading to the effective immunological memory required for good vaccine efficacy.

H5N1 continues to circulate and diversify [2] and some mutations accrued since 2004 may enhance the virus' capacity for receptor switching [21]. It is also noteworthy that lesser studied subtypes of avian influenza may be evolutionarily closer to receptor switching than previously assumed [11], [12], [41]. Tissue binding assays such as those described here will be useful in assessing the pandemic potential of such viruses. The widespread preparedness for an influenza pandemic that has resulted from the prevalence of H5N1 therefore remains appropriate even in light of the data we present.

## Materials and Methods

### Expression of influenza HA protein in insect cells

*Spodoptera frugiperda* cells (Sf9) were grown in suspension in BioWhittaker Insect-Xpress serum-free media (Cambrex) supplemented with 2% fetal calf serum. They were infected with recombinant baculovirus expressing H3-Fc, H5-Fc or H5-Fc receptor binding mutants and incubated at 28°C. After 72 h the supernatant was harvested by centrifugation at 4000 rpm for 10 min to remove the infected cells. The soluble protein was concentrated by centrifugation at 4500 rpm for 4 h using Vivaspin columns (Viva Science, Sartorius Group). The yield of concentrated protein ranged from 500–700 µg/mL. Protein was aliquoted and stored at −80°C.

### Binding of HA-Fc proteins to human airway epithelial culture and tracheal airway tissue sections

Paraffin embedded sections of cultured human airway epithelium or normal human tracheal sections were obtained from UNC Cystic Fibrosis Center Core facility. HAE derived from at least 3 codes and tracheal sections from 3 individuals were used in this study and representative images are shown. There was no notable difference between cultures or sections from different individuals. The sections were deparaffinized and rehydrated. Non-specific binding was blocked using 3% bovine serum albumin (BSA) in phosphate-buffered saline (PBS) for 1 hour at room temperature (RT) before incubation with ligands. Concentrated baculovirus supernatants containing H3, H5 or H5 HA and mutant proteins were pre-complexed with goat anti-human IgG (Fc specific) antibody (Invitrogen) at a ratio of 5 µg of HA-Fc: 0.6 µg of antibody, for 1 hour at 4°C. Sections were incubated with pre-complexed HA-Fc in 1% BSA in PBS ON at 4°C or RT for 3 hours. After washing in PBS-T, slides were incubated with rabbit anti-acetylated α-tubulin in 1% BSA in PBS for 3 hours RT or overnight (ON) at 4°C. Following washing with PBS-T (PBS with 0.05% Tween-20), antibody-incubated slides were immersed in 1% BSA in PBS containing donkey anti-goat FITC (Immunologicals Direct) and donkey anti-rabbit Alexafluor 647 (Invitrogen) for 1 hour at RT. Slides were washed with PBS-T and viewed under a confocal microscope. Images were collected on a Zeiss Pascal LSM5 laser scanning microscope using Axioplan2 imaging software using a Plan-Apochromat 63×1.4 oil Ph3 lens. The binding of each mutant to the different cell types was quantified by counting at least five different fields of view containing a total of 100–200 cells.

### Sialidase treatment of tissue sections

Five units of α2-3 sialic acid-specific neuraminidase from *Salmonella typhimurium* or 15 units of a α2-3/α2-6 dual specificity enzyme from *Clostridium perfingens* were incubated with deparaffinized sections for 3 hours at 37°C. Sections were then washed in PBS-T before being probed with recombinant HA proteins as above.
